# Management of Abnormal Uterine Bleeding Among Reproductive Age Group Women: A Cross-Sectional Study

**DOI:** 10.3390/jcm13237086

**Published:** 2024-11-23

**Authors:** Rina Abdullah Almuhaitb, Rinad Hamad Alenazi, Rauof Ahmad Almebki, Raghad Awadh Alshehri, Monya Mohammed Alemad, Joud Mohammed AlHarbi, Shahad Abdullah AlAmro, Renad Mohammed Alshahrani, Hanadi Bakhsh

**Affiliations:** 1 College of Medicine, Princess Nourah bint Abdulrahman University, Riyadh 11564, Saudi Arabia; renaabdullah@icloud.com (R.A.A.); 441002395@pnu.edu.sa (R.H.A.); 441004471@pnu.edu.sa (R.A.A.); 441003241@pnu.edu.sa (R.A.A.); 441004048@pnu.edu.sa (M.M.A.); 441003434@pnu.edu.sa (J.M.A.); 441000330@pnu.edu.sa (S.A.A.); 441001778@pnu.edu.sa (R.M.A.); 2 Obstetrics and Gynecology Department, College of Medicine, Princess Nourah bint Abdulrahman University, Riyadh 11564, Saudi Arabia; 3 Department of Obstetrics and Gynecology, King Abdullah bin Abdulaziz University Hospital, Princess Nourah bint Abdulrahman University, Riyadh 11564, Saudi Arabia

**Keywords:** abnormal uterine bleeding, PALM-COEIN classification, reproductive health, hormonal treatment, ovulatory dysfunction

## Abstract

**Background:** Abnormal uterine bleeding (AUB) is a common gynecological complaint affecting women of reproductive age. This study aimed to explore the management of AUB using the FIGO PALM-COEIN classification system. **Methods:** A cross-sectional study was conducted at King Abdullah bin Abdulaziz University Hospital, reviewing 500 medical records of women aged 20–50 years with AUB. Data on demographics, clinical characteristics, PALM-COEIN classification, and treatment modalities were collected and analyzed. **Results:** The majority of participants were aged 20–29 years (43%) and overweight or obese (64.2%). Ovulatory dysfunction (31.6%) was the most common identifiable cause of AUB, followed by leiomyoma (16.8%). Hormonal treatments, particularly combined oral contraceptive pills, were associated with improved outcomes (OR = 2.15, *p* < 0.001) and reduced anemia prevalence (*p* = 0.042). Age (OR = 0.95, *p* = 0.015) and BMI (OR = 1.10, *p* = 0.005) were significant predictors of treatment response. The presence of leiomyoma decreased the odds of treatment success (OR = 0.55, *p* = 0.007), while ovulatory dysfunction increased the likelihood of response (OR = 1.75, *p* = 0.003). **Conclusions:** The study highlights the complex nature of AUB and the effectiveness of hormonal treatments in its management. Findings emphasize the need for individualized treatment approaches based on the underlying etiology and patient characteristics. Future research should focus on long-term outcomes and optimizing management strategies for complex cases.

## 1. Introduction

Abnormal uterine bleeding (AUB) is a common gynecological condition affecting women during their reproductive years. It encompasses any deviation from the normal menstrual cycle, including changes in regularity, frequency, duration, or volume of menstrual flow that persist for more than 6 months in non-pregnant women [1]. Globally, AUB accounts for a significant proportion of gynecological consultations, with up to 30% of women experiencing this issue at some point in their lives [2]. Given its prevalence and impact, it is crucial to understand the underlying causes, clinical presentations, and treatment strategies to mitigate its effects on women’s health and quality of life [3].

AUB manifests in various forms, including menorrhagia (excessive menstrual bleeding), metrorrhagia (bleeding between periods), and polymenorrhea (frequent menstrual cycles) [4,5]. The chronic nature of these conditions can lead to significant physical, emotional, and social consequences for affected women [6,7]. Due to the substantial impact of AUB on quality of life, timely and effective medical intervention is essential [8].

The International Federation of Gynecology and Obstetrics (FIGO) developed the PALM-COEIN classification system to standardize the diagnosis and treatment of AUB [9]. This system categorizes AUB into structural causes (PALM: Polyps, Adenomyosis, Leiomyomas, Malignancy/hyperplasia) and non-structural causes (COEIN: Coagulopathy, Ovulatory dysfunction, Endometrial reasons, Iatrogenic factors, and Not yet classified) [10]. The adoption of this classification has significantly improved diagnostic accuracy and treatment decisions for AUB [11].

The etiology of AUB varies by factors such as age, parity, and comorbidities [12]. For example, structural causes like leiomyomas and polyps are more prevalent in older women, particularly those nearing menopause, while non-structural causes, such as ovulatory dysfunction, are more common in younger women and may be associated with endocrine disorders like polycystic ovarian syndrome (PCOS) [13,14]. Understanding the distribution of these causes is key to tailoring treatment strategies for individual patients [15].

AUB also poses significant health risks beyond menstrual irregularities. Chronic and severe bleeding can lead to iron deficiency anemia, manifesting as fatigue, weakness, and cognitive impairment [16]. In extreme cases, significant blood loss can result in hypovolemia, necessitating immediate medical attention [17]. Additionally, the unpredictable and distressing nature of AUB can have profound psychological effects, including anxiety, depression, and social isolation, which are often exacerbated by societal stigmas surrounding menstrual disorders [18].

To effectively manage AUB, a comprehensive approach that addresses both the underlying causes and the associated symptoms is required. Treatment options range from hormonal therapies, such as oral contraceptives and intrauterine systems (IUS), to non-hormonal options like NSAIDs and antifibrinolytics, and in more severe cases, surgical interventions such as endometrial ablation or hysterectomy [19]. The choice of treatment is influenced by factors such as the patient’s reproductive goals, comorbid conditions, and symptom severity [20]. The FIGO PALM-COEIN classification provides a valuable framework for clinicians to make informed treatment decisions, although challenges remain, as treatment efficacy can vary, and symptoms may persist or recur despite intervention [21].

Despite the availability of effective treatments, AUB often goes undiagnosed and untreated, particularly in resource-limited settings. Many women avoid seeking medical care due to limited awareness and cultural taboos surrounding menstruation [22]. Delayed treatment can exacerbate underlying conditions, leading to complications such as severe anemia or endometrial hyperplasia, which may increase the risk of developing endometrial cancer [23]. Therefore, raising awareness and promoting timely medical evaluation is crucial to improving health outcomes for women affected by AUB.

Endometriosis, a chronic gynecological condition characterized by the presence of endometrial-like tissue outside the uterus, is an important but often under-recognized cause of abnormal uterine bleeding (AUB) [24]. Although it is less prevalent compared to other structural causes such as leiomyomas or polyps, endometriosis significantly impacts reproductive-aged women by contributing to menstrual irregularities and chronic pelvic pain [1]. Its complex pathophysiology and association with other conditions like infertility make it an important factor to consider in the management of AUB [25].

### 1.1. Aim of the Study

The aim of this study is to explore the management of abnormal uterine bleeding (AUB) among women of reproductive age using the FIGO PALM-COEIN classification system. The study seeks to define the sociodemographic and obstetric profiles of these women, identify the underlying causes of AUB, evaluate the treatment patterns employed, and assess the complications associated with AUB. Ultimately, this research aims to enhance the understanding of AUB management in a clinical setting, contributing to improved patient outcomes and quality of care.

### 1.2. Research Question

What are the prevalent causes of abnormal uterine bleeding (AUB) among women of reproductive age, and how are these causes managed using the FIGO PALM-COEIN classification system in a clinical setting?

## 2. Materials and Methods

### 2.1. Research Design

This study employed a cross-sectional observational design to explore the management of abnormal uterine bleeding (AUB) among women of reproductive age. A cross-sectional design was chosen because it allows for the collection of data at a single point in time, providing a snapshot of the prevalence and management of AUB within the study population. This design is particularly suitable for identifying associations between variables, such as the causes of AUB and the treatment modalities used, without requiring long-term follow-up.

### 2.2. Setting

The study was conducted at the Obstetrics and Gynecology Department of King Abdullah bin Abdulaziz University Hospital (KAAUH), a secondary care facility located in Saudi Arabia. KAAUH is a well-equipped medical center that serves a diverse population, providing comprehensive gynecological and obstetrical care. The hospital is affiliated with a major academic institution, which facilitates research and access to a large patient population. The data collection period spanned from January 2024 to April 2024.

### 2.3. Sample and Sampling

#### 2.3.1. Sample

The sampling process employed in this study was systematic, aimed at ensuring that all eligible cases of abnormal uterine bleeding (AUB) within the defined data collection period were included. Rather than selecting patients based on specific demographic characteristics, the study systematically reviewed medical records of all women aged 20–50 years diagnosed with AUB at KAAUH.

To ensure representation across various demographic factors (such as age, BMI, and parity), no exclusion criteria were applied beyond the predefined inclusion criteria. All eligible patients who met the diagnostic and inclusion criteria were included in the study. This approach helped minimize selection bias, as all relevant cases during the specified time frame were reviewed without any deliberate selection process.

##### Inclusion and Exclusion Criteria

During the study period, a total of 750 women with a clinical diagnosis of abnormal uterine bleeding (AUB) were treated at the Obstetrics and Gynecology Department of King Abdullah bin Abdulaziz University Hospital. From this population, 500 women met the inclusion criteria and were included in the study. The remaining 250 patients were excluded due to factors such as incomplete medical records, pregnancy-related bleeding, or concurrent medical conditions that did not allow for a clear classification using the FIGO PALM-COEIN system.

The inclusion criteria for the study were:

- Age between 20 and 50 years.
- A clinical diagnosis of AUB based on the FIGO PALM-COEIN classification system.
- Non-pregnant status.

The exclusion criteria included:

- Pregnant women.
- Women with AUB due to gestational or postpartum conditions.
- Incomplete or unavailable medical records.

#### 2.3.2. Sampling

The sampling process for this study was purposive, non-probability sampling. This technique was chosen because it allows for the deliberate selection of cases that meet the specific inclusion criteria, thereby focusing on the most relevant cases for the research objectives. Given that the study aimed to explore AUB management within a defined patient population, purposive sampling was appropriate for ensuring that only cases with complete and relevant data were included.

The sampling process began with the identification of eligible patients. The research team used the TrakCare^®^ (InterSystems Corporation, Cambridge, MA, USA) electronic health record (EHR) system at KAAUH to generate a list of patients who met the inclusion criteria. This list was compiled by searching for women aged 20–50 who had been diagnosed with AUB and had visited the Gynecology clinic during the study period. Once the list of eligible patients was generated, the research team manually reviewed each medical record to confirm the diagnosis of AUB and to ensure that all necessary data were available. This step was crucial for verifying the completeness and accuracy of the information before including a patient in the study.

The sample size of 500 was determined based on statistical considerations, including a 95% confidence level and a margin of error that would allow for meaningful analysis. The chosen sample size was intended to provide sufficient power to detect significant associations between variables and to ensure the generalizability of the findings within the context of the study setting. For each selected patient, data were extracted using a structured data collection form. This form was designed to capture all relevant variables, including demographic information, clinical history, menstrual cycle characteristics, causes of AUB, treatment modalities, and associated complications.

Only pelvic or uterine surgeries directly relevant to the management of abnormal uterine bleeding (AUB), such as fibroid removal, hysteroscopy, or dilation and curettage (D&C), were included. Surgeries unrelated to reproductive health, such as strabismus correction, were excluded from analysis to maintain focus on interventions impacting AUB outcomes.

Data on participants’ prior surgeries, abortion history, and pre-term deliveries were extracted from medical records, providing insight into relevant obstetric and gynecological history. However, specific details regarding the method of prior deliveries (e.g., cesarean versus vaginal) were not available, as this level of detail was either not captured by the data system or fell outside the scope of the current study’s focus.

Although the sampling method was non-probability, the research team made efforts to ensure that the sample was representative of the broader population of women with AUB at KAAUH. This was achieved by including a wide range of patients across different age groups, BMI categories, and other relevant demographic factors. Additionally, efforts were made to avoid any selection bias by systematically including all eligible cases within the defined time frame.

##### Data Collection Tools

Data were collected using a structured data collection form specifically designed for this study. The form was developed by the research team and included sections on demographic information, obstetric and gynecological history, menstrual cycle characteristics, impact on quality of life, causes of AUB, related symptoms, past surgical history, investigations, treatments, and complications. The form was pre-tested on a small subset of records to ensure clarity and comprehensiveness.

Data on prior surgical history were reviewed with a focus on pelvic and uterine surgeries pertinent to AUB management. Non-reproductive surgeries (e.g., surgeries for strabismus or unrelated conditions) were excluded to ensure that the analysis centered on procedures with direct implications for reproductive health and AUB management.

The primary data source was the TrakCare^®^ system, an electronic health record (EHR) system used at KAAUH. This system allowed for the efficient extraction of relevant data from patient medical records. The research team manually reviewed each record to ensure accuracy and completeness of the data collected.

##### Procedure

The research team followed a systematic procedure for data collection. First, a list of eligible patients was generated from the TrakCare^®^ system based on the inclusion and exclusion criteria. Each patient’s medical record was then accessed, and the relevant data were extracted using the structured data collection form. The data were initially recorded in paper form and then entered into an Excel spreadsheet for further analysis.

Throughout the data collection process, the research team ensured consistency by adhering to a standardized approach for reviewing records and recording data. Regular team meetings were held to address any ambiguities or challenges encountered during data extraction. Once data collection was complete, the dataset was cleaned and prepared for analysis, with any discrepancies resolved through consensus.

##### Data Analysis

Data analysis was conducted using SPSS version 25 software. Descriptive statistics were used to summarize the demographic and clinical characteristics of the study population. Categorical variables, such as causes of AUB and treatment modalities, were presented as frequencies and percentages. Continuous variables, such as age and BMI, were presented as means and standard deviations.

The association between different causes of AUB (as classified by the PALM-COEIN system) and demographic variables was assessed using the Chi-square test for categorical data. A *p*-value of less than 0.05 was considered statistically significant. Subgroup analyses were also performed to explore differences in AUB management based on age groups, parity, and other relevant factors.

##### Ethical Considerations

Ethical approval for this study was obtained from the Institutional Review Board (IRB) at Princess Nourah bint Abdulrahman University (IRB No. 22-1047, approval date: 7 December 2022). The study was conducted in accordance with the ethical principles outlined in the Declaration of Helsinki. Given that the study involved the review of existing medical records, informed consent from individual patients was not required; however, patient confidentiality was strictly maintained.

All data were anonymized before analysis to protect patient privacy. Identifiers such as patient names, medical record numbers, and other personal information were removed from the dataset. Access to the data was restricted to the research team, and all electronic files were stored on password-protected computers.

## 3. Results

Key Terms:

1. Abortion: Add a sentence to clarify the term “abortion” in the study results. For example:
  ○ “In this study, ‘abortion’ refers to both spontaneous miscarriage and any type of pregnancy loss, as documented in the patients’ medical records.”
2. Pre-term: Clarify what “pre-term” refers to by specifying its meaning in the context of the study. For example:
  ○ “Pre-term in this study refers to a history of pre-term birth, defined as childbirth occurring before 37 weeks of gestation.”
3. Thyroid Conditions and PCOS: Provide explicit diagnostic criteria for hypothyroidism, hyperthyroidism, and PCOS, including relevant laboratory values. For example:
  ○ “Hypothyroidism was diagnosed based on elevated thyroid-stimulating hormone (TSH) levels (>4.0 mIU/L) and/or reduced free thyroxine (T4) levels, in accordance with standard diagnostic criteria. Hyperthyroidism was defined as a TSH level below 0.4 mIU/L with elevated free T4 or free triiodothyronine (T3). Polycystic Ovary Syndrome (PCOS) was diagnosed based on the Rotterdam criteria, which include the presence of at least two of the following: oligo- or anovulation, clinical or biochemical signs of hyperandrogenism, and polycystic ovaries on ultrasound.”

BMI Definitions:

Underweight: BMI < 18.5,
Normal weight: BMI 18.5–24.9,
Overweight: BMI 25–29.9,
Obesity: BMI ≥ 30.

The sample characteristics presented in Table 1 provide a comprehensive overview of the demographic and clinical profiles of 500 women with abnormal uterine bleeding (AUB). The majority of the participants were aged 20–29 years (43%), followed by those aged 40–50 years (30.2%). Most participants were either overweight (30%) or obese (34.2%), highlighting a significant prevalence of high BMI among the sample. Nearly half of the women were married (47.8%), with a substantial proportion being nulliparous (58.6%). The majority had regular menstrual cycles (67%), with heavy bleeding reported by 34% of the women. Although a small percentage of participants reported impacts on their social (3.8%) and emotional life (7.6%), a considerable number sought healthcare for related issues (26.6%).

Table 2 provides a comprehensive overview of the health status of 500 women with abnormal uterine bleeding (AUB). Notably, 11% of the participants had hypothyroidism, while a smaller proportion (1.8%) had hyperthyroidism. Hyperprolactinemia was observed in 3.6% of the women, and a significant 36.8% were diagnosed with Polycystic Ovary Syndrome (PCOS), highlighting the common comorbidities associated with AUB. Regarding surgical history, nearly a quarter (24.6%) had undergone previous surgeries. Symptoms such as fatigue, pelvic pain, and mood changes were present in 12.4%, 10.4%, and 7.6% of the participants, respectively. Among complications, anemia was prevalent in 32.6% of the women, while infertility affected 7.6%.

The data presented in Table 3 provide a comprehensive overview of the distribution of causes of abnormal uterine bleeding (AUB) among the 500 participants, classified according to the PALM-COEIN system. Structural causes (PALM) are less prevalent, with leiomyoma being the most common structural cause, affecting 16.8% of the sample. Polyps, adenomyosis, and malignancy/hyperplasia were identified in 3.8%, 1%, and 0.6% of cases, respectively. In contrast, non-structural causes (COEIN) are more prevalent, particularly ovulatory dysfunction, which accounts for 31.6% of cases. The most significant category, however, is the “Not yet classified” group, which includes 43% of cases, indicating a substantial proportion of AUB causes that remain undetermined by current diagnostic methods. Among the participants with a coagulopathy diagnosis, general coagulopathy status was recorded. However, specific types of coagulopathies, such as von Willebrand disease or platelet function disorders, were not consistently documented across medical records. This limitation restricts the ability to analyze coagulopathy subtypes within the study population.

The data presented in Table 4 illustrate the distribution of hormonal and non-hormonal treatments across different PALM-COEIN classifications of abnormal uterine bleeding (AUB) in a cohort of 498 women. The majority of patients across all categories were managed without the use of hormonal treatments, particularly in cases of malignancy and adenomyosis where 100% and 60%, respectively, did not receive hormonal therapy. For those who did receive hormonal treatment, the use of combined oral contraceptive pills (COCPs) was most common in ovulatory dysfunction (17.7%) and leiomyoma (11.9%). In terms of non-hormonal treatment, NSAIDs were the most frequently used, especially in cases of coagulopathy (37.5%) and leiomyoma (17.9%). Fibrinolytic treatment, specifically with tranexamic acid, was utilized in managing heavy menstrual bleeding among patients in this cohort. Tranexamic acid, an antifibrinolytic agent, helps reduce menstrual blood loss by inhibiting the breakdown of fibrin clots.

The results in Table 5 highlight significant associations between hormonal treatment and certain complications of abnormal uterine bleeding (AUB). Specifically, hormonal treatment is significantly associated with a reduced prevalence of anemia (*p* = 0.042) and an increased prevalence of mood changes (*p* = 0.015) compared to those who did not receive hormonal treatment. However, the associations between hormonal treatment and other complications, such as infertility (*p* = 0.119) and pelvic pain (*p* = 0.067), were not statistically significant.

The logistic regression analysis presented in Table 6 highlights several key predictors of response to treatment for abnormal uterine bleeding (AUB). Age is negatively associated with treatment response, with an odds ratio (OR) of 0.95, indicating that younger women are more likely to respond to treatment (*p* = 0.015). BMI is positively associated with treatment response (OR = 1.10, *p* = 0.005), suggesting that higher BMI increases the likelihood of a positive response. The presence of leiomyoma significantly decreases the odds of responding to treatment (OR = 0.55, *p* = 0.007), whereas ovulatory dysfunction is associated with a higher likelihood of response (OR = 1.75, *p* = 0.003). Hormonal treatment significantly enhances the probability of treatment success (OR = 2.15, *p* < 0.001), while non-hormonal treatment does not show a significant association (OR = 0.85, *p* = 0.350). Parity and the presence of polyps were not statistically significant predictors of treatment response.

## 4. Discussion

This cross-sectional study provides valuable insights into the management of abnormal uterine bleeding (AUB) among reproductive-age women using the FIGO PALM-COEIN classification system. The findings highlight the complex nature of AUB, its diverse etiologies, and the varied approaches to its management in a clinical setting.

### 4.1. Demographic and Clinical Characteristics

The study population predominantly consisted of younger women (43% aged 20–29 years), which is consistent with previous research indicating that AUB is a common gynecological complaint among women of reproductive age [26]. The high prevalence of overweight (30%) and obesity (34.2%) among participants aligns with existing literature suggesting a link between increased body mass index (BMI) and AUB [27]. This association may be attributed to the impact of excess adipose tissue on hormone metabolism, particularly estrogen, which plays a crucial role in endometrial regulation [28].

The majority of participants (67%) reported irregular menstrual cycles, with 34% experiencing heavy bleeding. These findings underscore the significant impact of AUB on women’s reproductive health and quality of life. Surprisingly, only a small percentage of women reported impacts on their social (3.8%) and emotional life (7.6%), which contrasts with some previous studies that have found more substantial psychosocial effects of AUB [29]. This discrepancy may be due to cultural differences in reporting or variations in the assessment of psychosocial impact [30,31,32].

### 4.2. Comorbidities and AUB

The high prevalence of Polycystic Ovary Syndrome (PCOS) (36.8%) among participants is noteworthy and consistent with existing literature that identifies PCOS as a common cause of anovulatory AUB [33]. The presence of thyroid disorders (11% hypothyroidism, 1.8% hyperthyroidism) and hyperprolactinemia (3.6%) further emphasizes the complex interplay between endocrine disorders and AUB. These findings highlight the importance of a comprehensive endocrine evaluation in women presenting with AUB, as addressing underlying hormonal imbalances may be crucial for effective management [34].

In this cohort, treatment approaches for AUB were primarily non-surgical, reflecting both institutional practices favoring conservative management and specific patient factors. Medical management, including hormonal therapies, was the mainstay of treatment, with no surgical interventions reported for the cases included. For cases of hyperplasia, diagnosis was confirmed through ultrasound imaging and biopsy findings, allowing for non-invasive assessment and classification. Where applicable, transvaginal ultrasound was employed to enhance diagnostic accuracy in identifying endometrial abnormalities.

### 4.3. PALM-COEIN Classification

The distribution of AUB causes according to the PALM-COEIN classification provides valuable insights into the etiology of AUB in this population. Non-structural causes, particularly ovulatory dysfunction (31.6%), were more prevalent than structural causes. This finding is consistent with other studies that have reported ovulatory dysfunction as a leading cause of AUB, especially in younger women [35].

The high percentage of cases classified as “Not yet classified” (43%) is noteworthy and highlights the challenges in definitively identifying the etiology of AUB in many cases. This underscores the need for continued research and refinement of diagnostic techniques to better categorize and understand the underlying causes of AUB [8].

Among structural causes, leiomyoma was the most common (16.8%), which aligns with previous studies identifying uterine fibroids as a significant contributor to AUB [36]. The relatively low prevalence of adenomyosis (1%) and endometrial polyps (3.8%) in this study may be due to limitations in diagnostic imaging or differences in the study population compared to other reports [37].

Although endometriosis was not the most frequent cause of AUB in our study, it remains a significant contributor to menstrual irregularities in women of reproductive age. Endometriosis-related AUB is often accompanied by dysmenorrhea and pelvic pain, complicating its clinical management [38]. Current treatment strategies for endometriosis-related AUB include hormonal therapies such as progestins, combined oral contraceptives, and GnRH agonists, which aim to suppress ectopic endometrial tissue growth and alleviate symptoms [39]. Surgical intervention, such as laparoscopy, may be required for more severe cases where medical management is insufficient [40].

The FIGO PALM-COEIN classification does not specifically account for endometriosis as a standalone category, though it may contribute to ovulatory dysfunction or other non-structural causes [41]. This underscores the need for comprehensive evaluation when managing AUB, particularly in women presenting with chronic pelvic pain or infertility, as these could be indicative of underlying endometriosis.

### 4.4. Treatment Modalities

The analysis of treatment modalities reveals a preference for non-hormonal management across most PALM-COEIN categories. This approach aligns with current guidelines that recommend starting with less invasive treatments before progressing to hormonal or surgical interventions [42]. The frequent use of NSAIDs, particularly in cases of coagulopathy and leiomyoma, is consistent with their established efficacy in reducing menstrual blood loss [43]. For hormonal treatments, combined oral contraceptive pills (COCPs) were the most commonly used, especially in cases of ovulatory dysfunction and leiomyoma. This preference for COCPs is supported by evidence of their effectiveness in regulating menstrual cycles and reducing menstrual blood loss [44]. The limited use of other hormonal options, such as the levonorgestrel intrauterine system (LNG-IUS), which has shown high efficacy in managing AUB [45], suggests potential areas for improvement in treatment strategies.

The logistic regression analysis revealed several important predictors of treatment response. The negative association between age and treatment response (OR = 0.95, *p* = 0.015) suggests that younger women may be more likely to respond favorably to AUB treatments. This finding could be related to the higher prevalence of ovulatory dysfunction in younger women, which often responds well to hormonal interventions [46].

The positive association between BMI and treatment response (OR = 1.10, *p* = 0.005) is intriguing and somewhat counterintuitive, as obesity is generally associated with poorer health outcomes. This finding warrants further investigation to understand the underlying mechanisms and potential implications for AUB management in women with higher BMI. The presence of leiomyoma was associated with decreased odds of treatment response (OR = 0.55, *p* = 0.007), which is consistent with the often challenging nature of managing AUB in women with uterine fibroids [47]. This highlights the need for tailored approaches and potentially more aggressive interventions in this subgroup of patients.

Ovulatory dysfunction, on the other hand, was associated with increased likelihood of treatment response (OR = 1.75, *p* = 0.003). This finding supports the efficacy of current management strategies, particularly hormonal treatments, in addressing anovulatory AUB [48]. The strong positive association between hormonal treatment and treatment response (OR = 2.15, *p* < 0.001) underscores the central role of hormonal interventions in AUB management. This finding is consistent with extensive literature supporting the use of hormonal therapies for various etiologies of AUB [49].

In recent years, the management of leiomyoma-associated abnormal uterine bleeding (AUB) has evolved with the introduction of newer hormonal treatments such as Ryeqo (relugolix-estradiol-norethindrone acetate) [50]. While our study primarily utilized more traditional hormonal therapies, including combined oral contraceptive pills and progestins, it is important to acknowledge the role of emerging treatments like Ryeqo, which has demonstrated efficacy in managing AUB, particularly in patients with leiomyoma [51]. Ryeqo combines a gonadotropin-releasing hormone (GnRH) antagonist with hormonal add-back therapy, offering an effective alternative for controlling heavy menstrual bleeding associated with fibroids, while minimizing hypoestrogenic side effects [52].

Although Ryeqo was not used in our cohort, it represents a promising option for patients, particularly those seeking non-surgical alternatives. Future studies should consider incorporating contemporary treatments like Ryeqo to evaluate their effectiveness compared to traditional methods in our patient population. This would allow for a more comprehensive approach to optimizing AUB management and improving patient outcomes.

### 4.5. Complications and Treatment

The association between hormonal treatment and reduced prevalence of anemia (*p* = 0.042) is an important finding, highlighting the potential of hormonal therapies to address one of the most significant complications of AUB [53]. However, the increased prevalence of mood changes associated with hormonal treatment (*p* = 0.015) emphasizes the need for careful monitoring and individualized treatment plans to balance efficacy with side effects [54]. The lack of significant association between hormonal treatment and infertility (*p* = 0.119) is reassuring, as it suggests that hormonal management of AUB may not substantially impact fertility outcomes. However, this finding should be interpreted cautiously, and further research is needed to fully understand the long-term effects of AUB treatments on reproductive outcomes [55].

### 4.6. Competition Section

The management of abnormal uterine bleeding (AUB) has been extensively studied, particularly in the context of using the FIGO PALM-COEIN classification system. Numerous studies have highlighted the effectiveness of hormonal therapies and surgical interventions for specific etiologies, including leiomyoma and ovulatory dysfunction. However, many of these studies either focus on smaller populations or do not adequately capture the broad spectrum of AUB causes categorized by the FIGO system.

In contrast, our study analyzes a larger cohort of 500 women, providing a comprehensive overview of both structural and non-structural causes of AUB. By applying the FIGO PALM-COEIN classification system, we were able to more accurately diagnose and evaluate treatment efficacy across a wider range of etiologies. Furthermore, our findings emphasize the importance of individualized treatment strategies, especially in the context of hormonal therapies, where responses varied significantly based on the underlying cause of AUB.

This study fills a critical gap by offering an extensive analysis of treatment outcomes in relation to the FIGO classification, particularly in a Middle Eastern context, where such data are limited. Our findings underscore the need for future research that continues to refine management strategies for AUB, particularly in addressing cases that remain “Not yet classified” by current diagnostic methods.

### 4.7. Limitations of the Study

While this study provides valuable insights into the management of AUB, there are several limitations. First, the cross-sectional nature of the study limits our ability to establish causal relationships between patient characteristics and treatment outcomes. A longitudinal design could provide more robust conclusions regarding the long-term efficacy of the treatments analyzed.

Additionally, the study was conducted at a single tertiary care center, limiting the generalizability of the findings to other settings. However, the results are applicable to populations with similar demographic and clinical characteristics. The high proportion of cases classified as “Not yet classified” also highlights limitations in the diagnostic process, suggesting that further refinement of the FIGO PALM-COEIN system is needed to address the full spectrum of AUB etiologies.

One notable limitation of this study is the absence of data regarding the delivery method, particularly cesarean sections. The influence of cesarean delivery on abnormal uterine bleeding (AUB) management remains a potential area of interest, as cesarean section history may affect uterine function or be associated with certain AUB etiologies such as adenomyosis or scarring. Future studies should incorporate cesarean section history to better assess its potential impact on AUB outcomes and management strategies.

While coagulopathy was noted as a contributing factor for AUB in some cases, specific diagnostic information (e.g., subtypes such as von Willebrand disease or platelet disorders) was not consistently available in the records. This limits the study’s ability to provide a detailed analysis of coagulopathy subtypes in relation to AUB management. Also, this study did not identify any cases involving uterine scar defects, commonly known as “niches”. It is unclear whether this was due to the absence of such cases within the study population or limitations in the documentation. Future research could benefit from explicit examination of uterine scar niches, as they may influence abnormal uterine bleeding in certain patients. This study did not include detailed classification of fibroids by type (e.g., submucosal, intramural, or subserosal), which limits our ability to analyze treatment outcomes based on specific fibroid characteristics. Future studies should consider incorporating fibroid classification to enhance the understanding of treatment responses associated with different fibroid types.

Finally, the study does not delve into the impact of endometriosis on AUB management, which is a known cause of the condition. Future studies should focus on this subgroup to broaden the understanding of AUB across diverse patient profiles.

### 4.8. Implications for Practice

This study contributes significantly to the understanding and management of AUB by providing region-specific insights into a population that has not been extensively studied: women of reproductive age in Saudi Arabia. The high prevalence of obesity and Polycystic Ovary Syndrome (PCOS) in this cohort highlights the critical need to tailor AUB management strategies to populations with similar sociodemographic profiles. Our findings also underscore the practical challenges of applying the FIGO PALM-COEIN classification system in clinical settings, particularly with the high percentage of cases classified as “Not yet classified”. This points to the need for enhanced diagnostic tools and methodologies, especially in populations with metabolic comorbidities.

Furthermore, the study presents important implications for future treatment practices. The data suggest that contemporary hormonal therapies, such as the use of combined oral contraceptives, can be highly effective, especially in managing AUB in women with ovulatory dysfunction. However, the findings also reveal a gap in the adoption of newer hormonal therapies, like Ryeqo, which should be considered in future management protocols.

## 5. Conclusions

This study offers novel insights into the management of AUB, particularly in populations with high rates of obesity and PCOS, conditions that are increasingly recognized as influencing both the prevalence and treatment response of AUB. Our findings demonstrate the importance of individualized treatment approaches that take into account the underlying comorbidities and sociodemographic factors unique to this population.

The high proportion of “Not yet classified” cases in the PALM-COEIN system raises important questions about the adequacy of current diagnostic frameworks for AUB and suggests a need for more comprehensive diagnostic tools. Moreover, the limited use of newer hormonal therapies like Ryeqo in practice represents an opportunity for future research and clinical innovation.

This study highlights the value of a systematic approach to AUB management using the FIGO classification framework. However, the high proportion of cases that remain “Not yet classified” underlines a critical need for improved diagnostic tools and methods to better understand and treat these unexplained cases. Tailoring treatment strategies to patient-specific factors, particularly in populations with prevalent metabolic and endocrine disorders, is vital to enhancing care quality and patient outcomes in AUB management.

By highlighting these region-specific challenges and opportunities for improving AUB management, this study not only contributes to the global literature but also underscores the need for further research tailored to populations with similar characteristics. The findings set the stage for developing more effective, individualized, and culturally relevant treatment protocols for women suffering from AUB.

## Figures and Tables

**Table 1:** Sample characteristics (*n* = 500).

Variable	Category	N (%)
Women’s age	20–29 years	215 (43%)
	30–39 years	134 (26.8%)
	40–50 years	151 (30.2%)
BMI	Underweight	18 (3.6%)
	Normal	138 (27.6%)
	Overweight	151 (30%)
	Obese	171 (34.2%)
	Unknown	22 (4.4%)
Marital status	Married	239 (47.8%)
	Single	219 (43.8%)
	Divorced	22 (4.4%)
	Widow	1 (0.2%)
	Unknown	19 (3.8%)
Parity	Nulliparous	293 (58.6%)
	≤3	122 (24.4%)
	>3	85 (17%)
Abortion	Yes	73 (14.6%)
	No	427 (85.4%)
Pre-term	Yes	44 (8.8%)
	No	456 (91.2%)
Regularity of menses	Regular	165 (33%)
	Irregular	335 (67%)
Cycle length	Frequent (<24 days)	71 (14.2%)
	Normal (24–38 days)	332 (66.4%)
	Infrequent (>38 days)	97 (19.4%)
Cycle duration	Prolonged (>8 days)	85 (17%)
	Normal (4–8 days)	386 (77.2%)
	Short (<4 days)	29 (5.8%)
Volume of blood	Heavy (>80 mL)	170 (34%)
	Normal (5–80 mL)	302 (60.4%)
	Light (<5 mL)	28 (5.6%)
Impact on social life	Yes	19 (3.8%)
	No	481 (96.2%)
Impact on emotional life	Yes	38 (7.6%)
	No	462 (92.4%)
Healthcare seeking for related health issues	Yes	133 (26.6%)
	No	367 (73.4%)

**Table 2:** Health status (*n* = 500).

Variable	Category	N (%)
Comorbidities	Hypothyroidism—Yes	55 (11%)
	Hypothyroidism—No	445 (89%)
	Hyperthyroidism—Yes	9 (1.8%)
	Hyperthyroidism—No	491 (98.2%)
	Hyperprolactinemia—Yes	18 (3.6%)
	Hyperprolactinemia—No	482 (96.4%)
	PCOS—Yes	184 (36.8%)
	PCOS—No	316 (63.2%)
Past surgical history	Yes	123 (24.6%)
	No	377 (75.4%)
Symptoms	Fatigue—Yes	62 (12.4%)
	Fatigue—No	438 (87.6%)
	Pelvic pain—Yes	52 (10.4%)
	Pelvic pain—No	448 (89.6%)
	Mood changes—Yes	38 (7.6%)
	Mood changes—No	462 (92.4%)
Complications	Anemia—Yes	163 (32.6%)
	Anemia—No	337 (67.4%)
	Infertility—Yes	38 (7.6%)
	Infertility—No	462 (92.4%)

**Table 3:** PALM-COEIN classification of causes (*n* = 500).

Category	Cause	Yes, N (%)	No, N (%)
PALM (Structural)	Polyps	19 (3.8%)	481 (96.2%)
	Adenomyosis	5 (1%)	495 (99%)
	Leiomyoma	84 (16.8%)	416 (83.2%)
	Malignancy and hyperplasia	3 (0.6%)	497 (99.4%)
COEIN (Non-structural)	Coagulopathy	8 (1.6%)	492 (98.4%)
	Ovulatory	158 (31.6%)	342 (68.4%)
	Endometrial	6 (1.2%)	494 (98.8%)
	Iatrogenic	2 (0.4%)	498 (99.6%)
	Not yet classified	215 (43%)	285 (57%)

**Table 4:** Treatment modalities in PALM-COEIN classification (*n* = 498).

Treatment Type		Polyps	Adenomyosis	Leiomyoma	Malignancy	Coagulopathy	Ovulatory	Iatrogenic	Endometrial	Not Yet Classified
Hormonal Treatment	No	13 (68.4%)	3 (60%)	58 (69%)	3 (100%)	6 (75%)	113 (71.5%)	1 (50%)	4 (66.7%)	167 (78.4%)
	IUS	0 (0%)	0 (0%)	3 (3.6%)	0 (0%)	1 (12.5%)	3 (1.9%)	0 (0%)	0 (0%)	1 (0.5%)
	COCP	4 (21.1%)	1 (20%)	10 (11.9%)	0 (0%)	0 (0%)	28 (17.7%)	0 (0%)	0 (0%)	29 (13.6%)
	IV Progesterone	0 (0%)	0 (0%)	1 (1.2%)	0 (0%)	0 (0%)	0 (0%)	0 (0%)	0 (0%)	0 (0%)
	Oral Progesterone	2 (10.5%)	1 (20%)	12 (14.3%)	0 (0%)	1 (12.5%)	11 (7%)	1 (50%)	2 (33.3%)	16 (7.5%)
	GnRH Agonist	0 (0%)	0 (0%)	0 (0%)	0 (0%)	0 (0%)	3 (1.9%)	0 (0%)	0 (0%)	0 (0%)
Non-Hormonal Treatment	No	18 (94.7%)	5 (100%)	59 (70.2%)	3 (100%)	4 (50%)	127 (80.4%)	1 (50%)	6 (100%)	162 (76.1%)
	Fibrinolytics	0 (0%)	0 (0%)	10 (11.9%)	0 (0%)	1 (12.5%)	13 (8.2%)	0 (0%)	0 (0%)	12 (5.6%)
	NSAIDs	1 (5.3%)	0 (0%)	15 (17.9%)	0 (0%)	3 (37.5%)	18 (11.4%)	1 (50%)	0 (0%)	39 (18.3%)

**Table 5:** Association between hormonal treatment and complications of AUB.

Complication	No Hormonal Treatment (N, %)	Hormonal Treatment (N, %)	*p*-Value
Anemia	85 (52.1%)	78 (47.9%)	0.042
Infertility	21 (55.3%)	17 (44.7%)	0.119
Pelvic Pain	29 (55.8%)	23 (44.2%)	0.067
Mood Changes	15 (39.5%)	23 (60.5%)	0.015

**Table 6:** Logistic regression analysis predicting response to treatment of AUB.

Variable	Odds Ratio (OR)	95% Confidence Interval (CI)	*p*-Value
Age (years)	0.95	0.92–0.99	0.015
BMI	1.10	1.03–1.17	0.005
Parity (Nulliparous)	1.25	0.87–1.80	0.230
Leiomyoma (Presence)	0.55	0.35–0.85	0.007
Polyp (Presence)	1.30	0.70–2.40	0.400
Ovulatory Dysfunction	1.75	1.20–2.60	0.003
Hormonal Treatment	2.15	1.50–3.10	<0.001
Non-Hormonal Treatment	0.85	0.60–1.20	0.350

## Data Availability

Data available upon request.
